# Building a sharable literature collection to advance the science and practice of implementation facilitation

**DOI:** 10.3389/frhs.2024.1304694

**Published:** 2024-05-09

**Authors:** Mona J. Ritchie, Jeffrey L. Smith, Bo Kim, Eva N. Woodward, JoAnn E. Kirchner

**Affiliations:** ^1^ VA Behavioral Health Quality Enhancement Research Initiative (QUERI), Central Arkansas Veterans Healthcare System, North Little Rock, AR, United States; ^2^ Department of Psychiatry, University of Arkansas for Medical Sciences, Little Rock, AR, United States; ^3^ Center for Healthcare Organization and Implementation Research, VA Boston Healthcare System, Boston, MA, United States; ^4^ Department of Psychiatry, Harvard Medical School, Boston, MA, United States; ^5^ VA Center for Mental Healthcare & Outcomes Research, Central Arkansas Veterans Healthcare System, North Little Rock, AR, United States

**Keywords:** implementation facilitation, practice facilitation, literature collection, implementation science, implementation strategies

## Abstract

**Background:**

Implementation science seeks to produce generalizable knowledge on *strategies* that promote the adoption and sustained use of evidence-based innovations. Literature reviews on specific implementation strategies can help us understand how they are conceptualized and applied, synthesize findings, and identify knowledge gaps. Although rigorous literature reviews can advance scientific knowledge and facilitate theory development, they are time-consuming and costly to produce. Improving the efficiency of literature review processes and reducing redundancy of effort is especially important for this rapidly developing field. We sought to amass relevant literature on one increasingly used evidence-based strategy, implementation facilitation (IF), as a publicly available resource.

**Methods:**

We conducted a rigorous systematic search of PubMed, CINAHL, and Web of Science citation databases for peer-reviewed, English-language articles with “facilitation” and a combination of other terms published from January 1996 to December 2021. We searched bibliographies of articles published from 1996 to 2015 and identified articles during the full text review that reported on the same study. Two authors screened 3,168 abstracts. After establishing inter-rater reliability, they individually conducted full-text review of 786 relevant articles. A multidisciplinary team of investigators provided recommendations for preparing and disseminating the literature collection.

**Findings:**

The literature collection is comprised of 510 articles. It includes 277 empirical studies of IF and 77 other articles, including conceptual/theoretical articles, literature reviews, debate papers and descriptions of large-scale clinical initiatives. Over half of the articles were published between 2017 and 2021. The collection is publicly available as an Excel file and as an xml file that can be imported into reference management software.

**Conclusion:**

We created a publicly accessible collection of literature about the application of IF to implement evidence-based innovations in healthcare. The comprehensiveness of this collection has the potential to maximize efficiency and minimize redundancy in scientific inquiry about this strategy. Scientists and practitioners can use the collection to more rapidly identify developments in the application of IF and to investigate a wide range of compelling questions on its use within and across different healthcare disciplines/settings, countries, and payer systems. We offer several examples of how this collection has already been used.

## 1 Introduction

Implementation science (IS) is the study of methods for promoting uptake of research findings and evidence-based practices (EBPs) into routine clinical practice to improve the quality and effectiveness of healthcare (1). Particularly, IS seeks to produce generalizable knowledge on specific *strategies* that promote the uptake and sustained use of EBPs and other clinical innovations. The rapid growth of this field in recent decades has involved combining expertise and lessons learned from multiple disciplines and areas of inquiry, including health sciences, organizational change, behavioral science, and systems engineering. As such, implementation science utilizes both interventional and observational study designs, employing quantitative, qualitative, and mixed methods approaches (2, 3).

To comprehensively understand the multidisciplinary and multi-method content of this growing field, recent work has been undertaken to develop compilations of relevant literature and methods used in implementation science. Examples include systematic reviews of how implementation-related theories, models, and/or frameworks are used (4, 5), as well as the Expert Recommendations for Implementing Change (ERIC) study that provided a comprehensive compilation of implementation strategy terms and definitions (6). For instance, since publication of the ERIC compilation, numerous advancements in implementation knowledge have stemmed from that work, including adapted strategy compilations specific to particular settings (7) and reviews of implementation strategies for specific clinical conditions (8). As such, these and other similar syntheses serve as cornerstones upon which research advancements can be built.

As implementation science advances, not only is there a continued need for these rigorous syntheses, but also accessible tools that make findings from such syntheses easily and widely available to implementation practitioners and scientists—i.e., tools that can maximize the syntheses' impact by enabling their findings and resources to be accessed and used without requiring large amounts of additional time and effort on the part of the user. A potential tool that can address this need is publicly available literature review findings on compelling and complex implementation science topics.

Because rigorously conducted literature reviews can advance scientific knowledge and facilitate theory development, they are increasingly being conducted. For example, the number of systematic reviews increased 20-fold from 2000 to 2019 (9). However, rigorous literature reviews are time consuming and can require significant scientific resources; thus, they are costly to produce (10, 11). Additionally, they are often narrowly focused within broader areas of study or practice. Improving the efficiency of literature review processes and reducing redundancy of effort is especially important for the rapidly developing field of implementation science. Literature reviews on specific implementation strategies, for example, can help us understand how they are conceptualized and applied in individual studies, synthesize findings across studies, and identify knowledge gaps to address in future research (6, 12–14).

One such strategy, implementation facilitation (IF), is multifaceted and involves at least one identified facilitator and a process of interactive problem-solving and support that occurs in a context of a recognized need for improvement and supportive interpersonal relationships (6). Typically, this strategy bundles an integrated set of activities to support uptake of evidence-based practices (EBPs) and other clinical innovations (15, 16). A growing body of literature shows IF to be an evidence-based strategy, particularly for implementing relatively complex EBPs; this evidence is robust across diverse clinical settings, including under-resourced, later-adopter locations (17–21).

Despite evidence of IF's impact, there remain gaps in knowledge concerning the application and evaluation of the strategy. For example, one gap is determining the “active ingredients” or mechanisms of multifaceted IF strategies and documenting mediators and moderators of IF effectiveness (22). Another gap is ascertaining the variety of skills and techniques to emphasize in the content and educational methods utilized in IF training/mentoring programs (23). Further, researchers are still unclear on the requisite composition and intensity of IF strategies for a given implementation effort based on varying contextual factors across settings, recipient characteristics, and/or complexity of the clinical innovation itself (24). To address these gaps, the field could benefit from innovative, non-traditional tools made readily available to enable more rapid and streamlined investigation into these and other important questions pertaining to IF strategies. For example, active learning groups such as the Department of Veterans Affairs (VA) Behavioral Health Quality Enhancement Research Initiative's (QUERI) Implementation Facilitation Learning Collaborative (23) and the North American Primary Care Research Group's practice facilitation community (25) could benefit from such tools in their work to identify and address gaps in the science and practice of IF. Below we describe the development of a comprehensive collection of literature on the conceptualization and application of IF strategies. A tool that may be useful to researchers in identifying gaps in IF knowledge/practice by investigating what is known on a given topic from the extant body of IF literature, knowledge which could then be used to inform planning and execution of new studies focused on addressing such gaps. Indeed, documenting what is known on a given research topic or gap in knowledge (e.g., background) and potential impact of proposed research if successful in addressing such gaps (significance) are routine components of grant proposals for new studies.

We created the IF literature collection using established systematic review methods; it is publicly available and comprehensive in its focus on IF across healthcare settings. Our goal was to amass relevant literature including empirical research, examples of clinical initiatives, conceptual articles, and theories regarding how IF supports implementation of EBPs for improving healthcare quality and/or delivery. Although many systematic and other types of reviews are conducted to answer a scientific question, this literature collection is unique in that it provides a rigorously vetted resource others may use to scan articles related to their own specific questions, such as existing gaps in knowledge about IF. We describe below the methods used to compile the literature collection and its features. We also share examples of how the collection has already been used to address important questions pertaining to the science and practice of IF. These examples highlight the advantages of this type of tool that relies on rigorous methods to identify relevant literature yet is not narrowed to any particular discipline or setting, allowing open access to researchers and practitioners beyond our team that wish to answer compelling questions about IF. The methods, results, and examples that we share demonstrate how such open access literature collections can optimize the yield of substantial time and effort invested in systematic literature searches that usually have to be replicated by different teams to answer different questions about related topics.

## 2 Methods

MJR and JLS, both implementation scientists for over twenty years, created the collection of IF literature in four phases, described below. MJR has extensive experience in evaluating IF strategies to support implementation of EBPs and other clinical innovations. JLS has extensive experience providing implementation facilitation to healthcare systems and in training and mentoring IF researchers and practitioners. Both MJR and JLS have led and contributed to development of multiple tools and resources that support IF research and practice (19, 26, 27). In the fourth phase (see below), additional VA Behavioral Health QUERI investigators, including JEK, BK, and ENW, provided consultation on preparing the collection for dissemination.

### 2.1 Phase 1: initial search of articles published 1996–2015

#### 2.1.1 Search strategy

We focused our search on articles about the *application* of IF as an implementation strategy; thus, our primary search term was the word, facilitation. Scholars have used different terminology to describe the process of providing support for implementing innovations in healthcare, e.g., implementation facilitation, practice facilitation, coaching, and technical assistance (28). Although we use the term implementation facilitation (IF) in this paper, we wanted the literature collection to be as inclusive as possible. To develop the search strategy, we identified key words in fifty articles about the application of facilitation for supporting implementation, regardless of the term used for the strategy by authors of those articles. We modified a previously developed search strategy for identifying papers focused on use of facilitation in nursing (29), adding key words from those other articles. Thus, our search strategy included the word “facilitation” and a combination of Medical Subject Heading (MeSH) terms and key words (see Supplementary File 1).

Additionally, IF has been applied in a variety of sectors. Because the organizational context varies widely across sectors, we elected to restrict the collection to articles about IF applied in healthcare and public health settings. Thus, our search strategy included three widely used bibliographic databases that index articles for those settings. With the assistance of a medical research librarian, we conducted an initial search of PubMed, Web of Science, and CINAHL citation databases on January 29, 2016 for English language articles published from January 1, 1996 through December 31, 2015. The concept of facilitation in healthcare is often traced back to the Oxford Prevention of Heart Attack and Stroke Project in England from 1982 to 1984 (30). However, the selection of the 1996 start date for our review was influenced by the emergence of implementation science as a field in the mid to late 1990's to address gaps between development of research evidence and its application in routine clinical practice (31–33).

#### 2.1.2 Article screening and selection

The medical research librarian imported the results of our search into RefWorks, a web-based citation management platform, and we identified and removed duplicate articles using RefWorks' deduplication feature. Next, we conducted a search on neurobiology and sports physiology terms that seemed most likely to be unrelated to implementation facilitation (including “synap,” “animal,” “physiology,” “cell,” and “neuro”); reviewed the titles and abstracts of identified records; and eliminated articles that were obviously irrelevant. We then imported the remaining citations into Covidence, a web-based software platform that streamlines the production of systematic reviews (34).

In the first stage of the screening process, the two reviewers (MJR and JLS) each independently screened all titles and abstracts to assess relevance and then discussed and resolved any conflicts. The second stage of the screening process consisted of a full text review of potentially eligible articles in which the two reviewers first independently reviewed ten percent of the articles for inter-rater reliability testing. The results showed a high level of agreement (96.8% agreement; Cohen's *κ* 0.93) on decisions regarding whether the article should be included/excluded for the literature collection. The remaining articles were then divided between the two reviewers for completion of the full text review process. When a study article mentioned other article(s) that were reporting on the same study, we conducted a full text review of the related article(s) and, when eligible for inclusion, added it/them to the collection.

The term, facilitation, has been used in multiple ways in scholarly literature, and facilitation to support implementation can be applied in any organizational setting. The types of interventions, i.e., practices, programs, and policies, needed for improvement vary across types of organizations and settings. We sought to focus this literature collection on the use of facilitation to support implementation in healthcare and public health settings. Thus, we defined IF as the use of a designated person or persons in an appointed role to support implementation of a new clinical practice, program, or policy. We excluded articles about facilitation that did not meet this definition and/or were not about the use of IF strategies in healthcare or public health settings. Additionally, we wanted to include scholarly, peer-reviewed journal articles that presented studies of implementation facilitation or contributed to our conceptual or theoretical understanding of this implementation strategy. We thus excluded dissertations, book reviews, editorials, and commentaries. We also excluded duplicate articles, and articles with insufficient information to determine their relevance; these are commonly used exclusion criteria. Given the diverse nature of the literature collection, we did not conduct a quality appraisal of the studies.

### 2.2 Phase 2: reference list search of articles

Because articles about the application of IF in healthcare were relatively novel during the early years of our phase 1 search, we examined the reference lists of articles published from 1996 through 2015 included in the literature collection. We added articles identified as potentially relevant to our Covidence database and repeated the two-stage screening process described above for phase 1.

### 2.3 Phase 3: updates to collection 2016–2021

To update the collection for 2016 through 2021, the medical research librarian periodically conducted searches using the same strategy except for the start and end dates of the search (see Table 1 for the dates when searches were completed). We followed the procedures described above for phase 1 for deduplication and screening of articles for inclusion in the literature collection.

**Table 1:** Search parameters and dates of completion.

Phase	Search	Search parameters	Search completed
1	Initial search	1 January 1996–31 December 2015	1 January 2016
2	Reference list search	1 January 1996–31 December 2015	19 September 2018
3	2016 update	1 January 2016–31 December 2016	7 August 2017
	2017 update	1 January 2017–31 December 2017	23 January 2018
	2018 update	1 January 2018–31 December 2018	14 June 2019
	2019–2020 update	1 January 2019–31 December 2020	15 June 2021
	2021 update	1 January 2021–31 December 2021	24 May 2022

### 2.4 Phase 4: preparation for dissemination

The reviewers presented an overview of the collection of articles and the methods described above to additional VA Behavioral Health QUERI investigators in three of their operational program team meetings. Investigators confirmed the value of the collection for advancing the science and practice of facilitation and discussed and made recommendations regarding who should have access to the collection, how the collection should be organized, how it should be disseminated, and what information should be disseminated along with the collection (e.g., the PRISMA chart). They also discussed mechanisms and parameters for identifying studies, study settings, and related studies included within the collection. Additionally, we identified categories of readily available information that might help users find articles of interest. To prepare the collection for dissemination, we imported records into EndNote reference management software. We then tagged records of articles with the type of article (e.g., study, conceptual/theoretical, IF literature review), study setting (e.g., USA, Canada), whether the study was conducted in a low- or middle-income country (LMIC), and the study acronym (if available). When multiple articles had been published for the same study, we listed all articles in a note record. During the preparation process, we discovered that keywords had been inconsistently imported between citation databases, and we imported available keywords from citation database records into EndNote records.

## 3 Results

**Figure 1:**
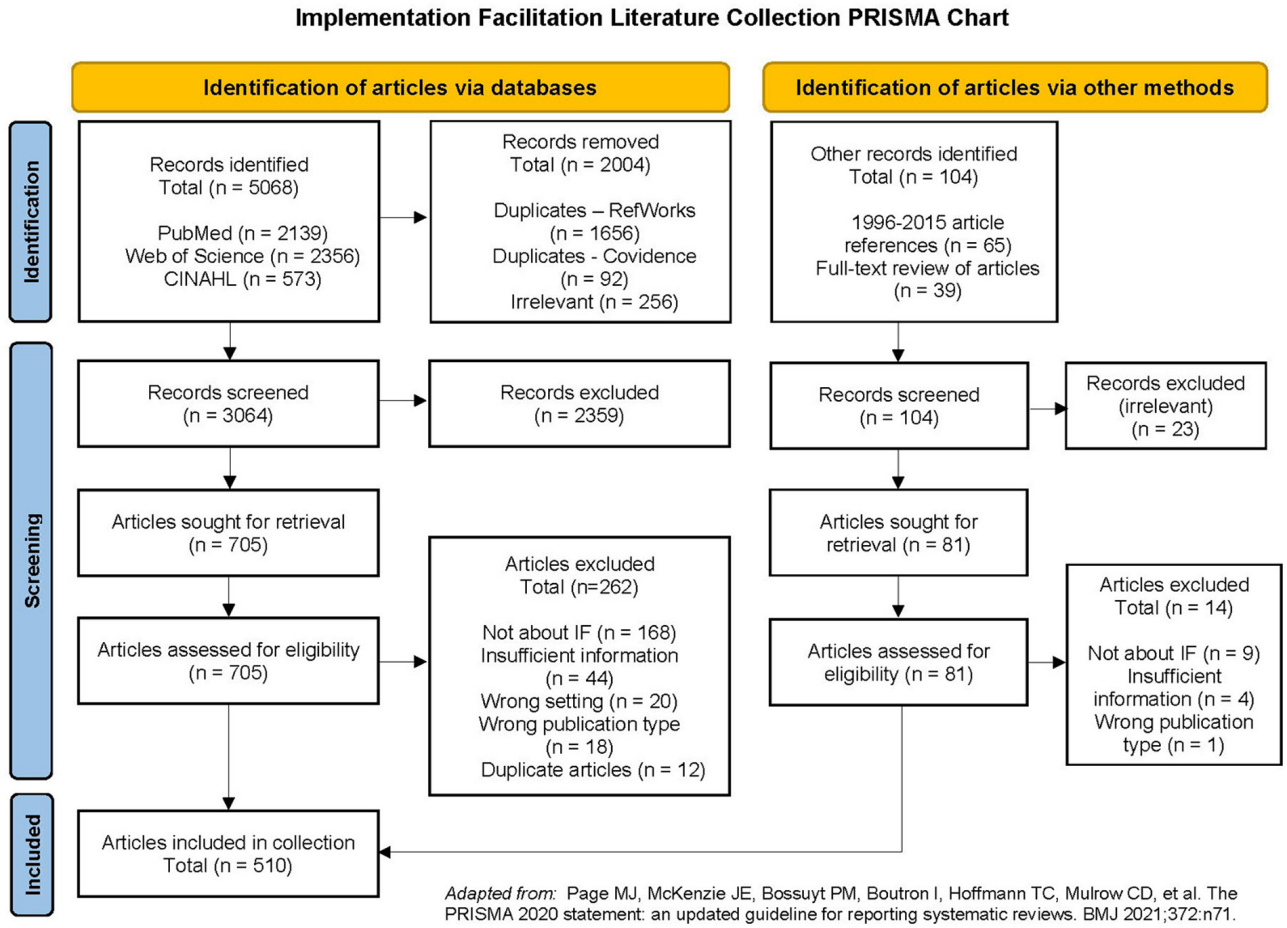
Implementation facilitation literature collection PRISMA chart.

To ensure the collection will be useful for conducting all types of reviews, we report the results of our search and screening processes for Phases 1–3 in accordance with the updated PRISMA (Preferred Reporting Items for Systematic Reviews and Meta-Analyses) guidelines (35–38) (see Figure 1). The literature search of bibliographic databases yielded 5,068 articles for screening. After eliminating 1,748 duplicates and 256 articles in a preliminary search on neurobiology and sport physiology terms, we screened titles and abstracts for 3,064 articles, rejecting another 2,359 articles that were irrelevant. We conducted a full text review of the remaining 705 articles, ultimately selecting 443 articles for inclusion in the collection. The majority of the articles excluded during full text review were found to be irrelevant because they were not about implementation facilitation. Our Phase 2 reference list search of articles from 1996 to 2015 and our full text review across all phases yielded an additional 104 articles for screening. After screening titles and abstracts and conducting full text review, we added 81 of those articles to the collection. In total, the collection is comprised of 510 articles.

**Figure 2:**
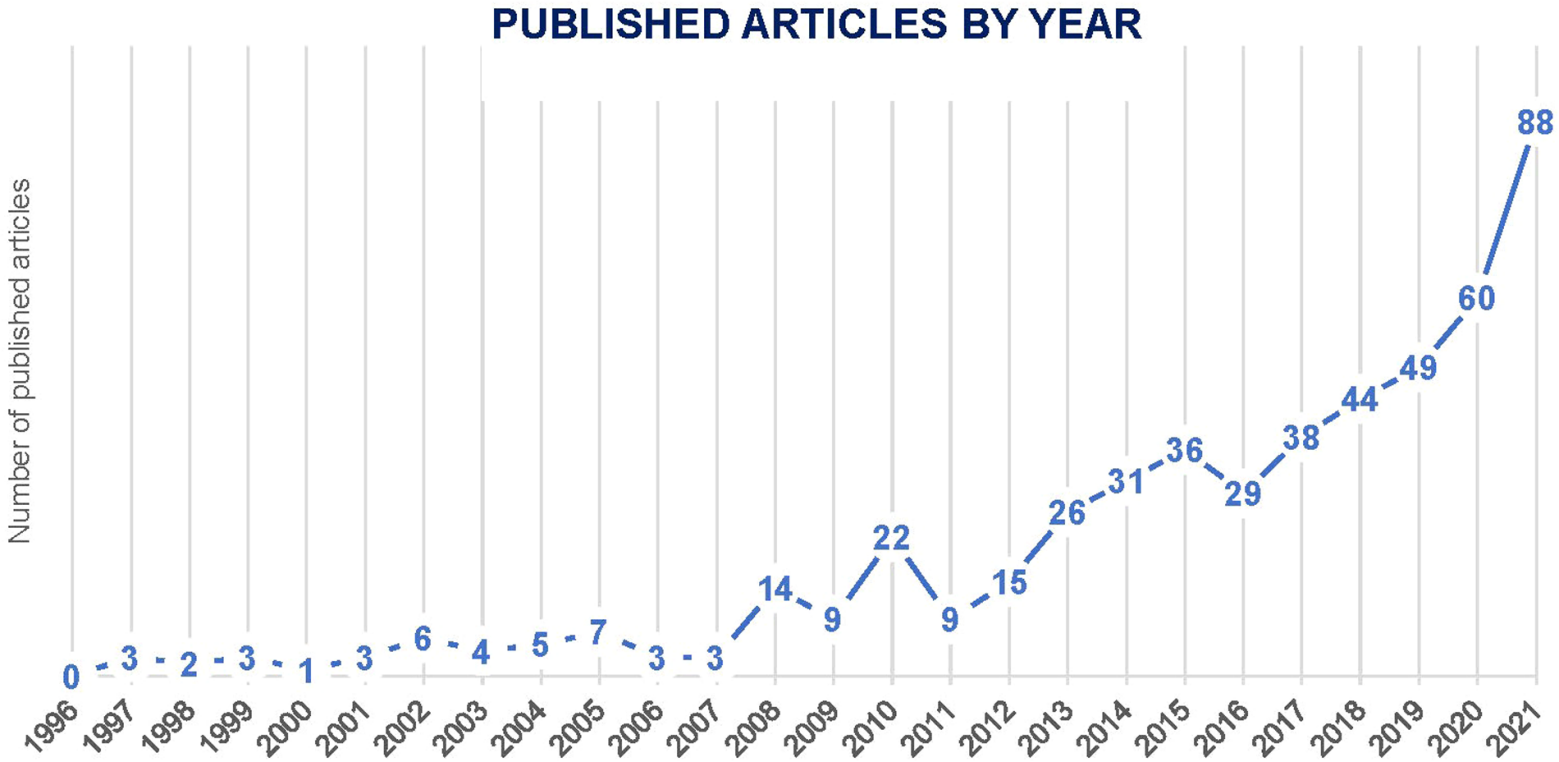
Number of included implementation facilitation-related articles published by year.

The literature collection includes 277 studies of implementation facilitation. Of these, there is only one published article for 203 of the studies; and there are from two to nine articles for each of the other 74 studies. The majority of the studies were conducted in the United States (*n* = 160), Canada (*n* = 28), United Kingdom (*n* = 25) and Australia (*n* = 24). Only ten studies were conducted in low- and middle-income countries. The collection also includes a variety of other articles (*n* = 77), e.g., conceptual/theoretical papers, literature reviews, debate papers, case studies, and descriptions of clinical initiatives. Articles in the collection were published in 168 different journals, and the number of articles published each year has steadily increased since 1996, with over half of the articles (54.7%) published from 2017 to 2021 (see Figure 2).

The implementation facilitation literature collection is available as an Excel file that includes a spreadsheet of study citations (research articles) and another spreadsheet of citations for non-study articles (see Supplementary File 2). Additionally, an xml file that can be imported into reference management software will be publicly available at: https://www.queri.research.va.gov/tools/implementation.cfm. In addition to keywords imported from citation databases, the keyword fields in these files include a word or phrase for the type of article (e.g., study, conceptual/theoretical); the study name or acronym when available; and the acronym, LMIC, when a study was conducted in a low- to middle-income country. The notes fields for each study article includes the location of the study (e.g., USA), and, when relevant, a list of other articles in the collection that report on the same study.

## 4 Discussion

This paper describes development of a comprehensive collection of literature, readily accessible for literature searches within and beyond our team, about the application of implementation facilitation for supporting use of evidence-based practices and programs in healthcare and public health settings. The collection includes peer-reviewed articles documenting empirical studies, conceptual and theoretical articles, and descriptions of large-scale implementation initiatives. To support users wishing to conduct systematic reviews to address questions about implementation facilitation, we applied and documented rigorous, established methods for the search and selection of articles included in the collection.

The primary goal of implementation science is to identify factors that affect the uptake of evidence-based practices and other clinical innovations in order to improve the quality and effectiveness of healthcare (31). The field emerged in response to the understanding that there was a significant gap between establishment of evidence for innovations (e.g., new therapies) and their uptake in routine clinical practice (39). In addition to studying the methods and strategies that promote uptake, investigators have created and/or fostered use of publicly available resources for other implementation *scientists* [i.e., free measure repositories (40)] and *practitioners* or those responsible for initiating change or increasing use of innovations in real-world settings [i.e., Learning Collaboratives and Communities of Practice (41)]. Such resources contribute to collective learning. For these reasons, we have developed and now present to the field an Implementation Facilitation Literature Collection as one such resource. The comprehensiveness of the collection and rigorous methods utilized in its creation have the potential to minimize redundancy in scientific inquiry about this implementation strategy, potentially offering greater efficiency in application of limited implementation science resources. By providing this literature collection, all parties will be better able to investigate important questions concerning IF strategies to more rapidly identify developments in its use and key lessons from both research- and practice-based initiatives. Moreover, due to the expansive scope of our collection, it can serve as a readily-accessible resource for investigating a wide range of important questions on the use of IF strategies within and across different healthcare disciplines/settings, countries (including developed as well as low-and-middle-income countries), and healthcare payer systems.

Indeed, our literature collection can be and is already being used to answer specific questions pertaining to the use of IF. For example, to transfer successful implementation strategies from research to practice, it is important to be able to measure and support fidelity to an implementation strategy's core activities (42). Unfortunately, this aspect of implementation science has been underdeveloped and infrequently applied (43). To address this gap, our literature collection was used in a comprehensive scoping review to (a) identify the range of activities reported in the use of IF strategies to support implementation of clinical innovations in healthcare settings, and then (b) inform deliberations within a rigorous, multi-stage expert panel consensus development process to identify core IF activities for different phases of implementation (19). Ultimately, the expert panel process, informed by the scoping review of this IF literature collection, identified a total of 16 core activities that can be used to assess fidelity to the IF strategy in research and practice applications. Without this collection, processes to extract data from relevant articles and then completing the expert panel process would have taken approximately 7–9 months longer to accommodate the development, refinement, and conduct of the article search and selection processes.

The literature collection was also used by ENW to conduct an environmental scan in the U.S. to identify examples of patient engagement in healthcare service delivery implementation activities (such as facilitation) (44). The research team's goal was to amass a body of examples of this work, synthesize challenges, and identify promising solutions to patient engagement in implementation activities, such as facilitation. The research team used the IF literature collection as one of the data sources for the scan, and because our collection was produced using rigorous systematic review methods, was well-documented, and readily accessible, it provided an efficient and trustworthy source of literature articles as data, saving the research team approximately 12–18 months of work (which, as for the previous use case, would have been necessary for comprehensive article search and selection). Although the environmental scan focused on U.S. examples of patient engagement in implementation activities, one interesting finding from our literature collection was that researchers reported it was the only data source that identified the most intensive levels of patient engagement in implementation activities that occurred in countries outside the U.S., such as Vietnam (45).

These examples of how the IF literature collection has been used underscore its ability to serve as a useful compendium of implementation-related knowledge that supports the advancement of implementation research and practice. And even if a future project that uses this collection occurs further from the timeframe that the collection covers, this collection will still save time for that project by requiring additional review only of relevant studies since the end of this collection's timeframe. Especially when considering that a majority of even the highly regarded Cochrane reviews are published more than two years after establishing their protocol (with more than 10% taking over five years) (46), we see the potential for this collection to remain useful for those studying implementation facilitation through the upcoming years.

While this particular compendium focuses on IF, there exist other such knowledge compendia that serve as firm bases upon which new implementation research and practice are being conducted. One example is the aforementioned compilation of implementation strategy terms and definitions, ERIC, which has supported the establishment of a common nomenclature and definitions for implementation strategies (6). Another example is the Consolidated Framework for Implementation Research (CFIR), which provides a curated collection of factors that have been found to affect implementation (47, 48). Initially developed in 2009 and updated in 2022, this framework is being actively integrated with systematic approaches to strategy design (49), including in combination with ERIC (50). A third example is the Consortium of Implementation Science's Implementation Science News, which provides searchable collections of up-to-date research, news, and opportunities for the implementation science community (51). Meaningful future contributions of this IF literature collection may result from potential collaborations with and linkages to these other knowledge compendia. For instance, we can work with their development and maintenance teams to explore the possibility of pointing researchers accessing their tools to this IF literature collection.

Such accessible and widely applicable compendia are especially important for bodies of knowledge that are rapidly growing, such as the field of implementation science. Their continued applicability is heavily dependent on being kept up to date, as is exemplified by each example compendium's refinements and/or regular updates that continue since their initial conceptualization and creation. This suggests that, for our IF literature collection to maintain its wide applicability over time, future updates and refinements are indispensable. While there is no standardized guidance on the appropriate cadence or frequency of such updates, two sources of information may be highly relevant to consider. First, the past decade has witnessed a rapidly growing number of living systematic reviews (52), which are systematic reviews that undertake continual updates to incorporate latest relevant research. Decisions regarding when and whether to update living systematic reviews are informed by relevant policy changes, publications that may alter an earlier review's conclusions, and feasibility of (e.g., available funding for) conducting an update (53), each of which can be considered in updating our IF literature collection. Second, the iterative process for refining previously developed products (54, 55), widely used in fields such as engineering and marketing, can be adapted to guide updates to our collection. For instance, the iterative process calls for an evaluation of how well a product is serving its intended purpose to inform decisions on whether to embark on an iteration of refinement. In this case, feedback from users of our collection on how they are finding the collection useful (and/or how not) could inform a data-driven decision regarding whether an update is necessary to ensure the collection's continued worth and applicability.

The strengths of our approach for developing the IF Literature Collection, as well as its potential utility and value to the field, are well-described herein; however, some limitations should be considered. First, our search was not fully inclusive of all bibliographic databases. Rather, we conducted our search in three widely used citation databases that index articles for healthcare and public health settings. We may have missed articles that were indexed in other less-commonly used citation databases, or which were not indexed in any database. Second, the collection includes articles published through December of 2021. The collection will need to be updated periodically in order to remain relevant and to maximize utility for the field. An example of such an ongoing updated review that is available online is the Patient-Centered Outcomes Research Institute's (PCORI) Engagement in Health Research Literature Explorer (56). Updates to this tool are made possible by PCORI staff members, and such consistent staffing is not often available for most reviews that are conducted, including ours. Further, the optimal and appropriate approaches for updating and replicating reviews are open questions that continue to be examined for systematic reviews at large (57, 58). As more knowledge is gained on that front, collections such as ours and the PCORI tool may benefit from incorporating the newest knowledge on best practices for updating reviews.

In this paper, we describe a publicly available collection of implementation facilitation literature and the rigorous methods used in developing it. There are a number of ways this foundational work could be further developed and enhanced. First, as with prior compilations such as the ERIC study (59), a clearly delineated path to update the collection over time and indication of where the responsibility lies to maintain the collection will be necessary if it is to remain relevant and useful long-term. As previously discussed, the collection could be regularly updated and evaluated; these processes would be facilitated by a supportive technical infrastructure and development of a defined procedure to protect the quality of the collection. To support further development, it will be critical to collect data on how this literature collection can be improved. Areas to consider include the content of the literature abstraction, sorting terms (metadata) readily available within the collection, and formats that can best serve distribution and use of the collection. Second, the collection is currently file-based and thus static. The collection could provide the foundation for a living collection posted on an interactive, web-based platform (e.g., the previously mentioned PCORI Literature Explorer). The infrastructure could thus be adapted and used to create literature collections on other implementation strategies. Third, the current collection includes most of the metadata downloaded from the online databases we searched with the addition of easily accessible descriptors, e.g., setting and type of article. Future work could incorporate additional metadata, e.g., characteristics of facilitation, theoretical model used, and payer system, thus, expanding the searchability of the collection. The literature collection and the rigorous methods used to create it can provide the foundation for further development. Although such work is beyond the capacity and resources of our team, we believe that the current collection will be valuable to implementation scientists and practitioners as they seek to understand what is currently known and unknown about IF. Additionally, the description of the established systematic review methods for article search and selection in this manuscript can provide the foundation for further development of the collection and represent a potential process for others who undertake the time-intensive task of creating literature collections. This is especially important for rapidly growing fields such as implementation science, particularly given the numerous discrete implementation strategies (6) that may be applied to support improved uptake of evidence-based practices and programs.

## Data Availability

The original contributions presented in the study are included in the article/**Supplementary Material**, further inquiries can be directed to the corresponding author.
