# Supplementary material for: Building a sharable literature collection to advance the science and practice of implementation facilitation
Source: Front Health Serv. 2024 May 9;4:1304694. doi: 10.3389/frhs.2024.1304694 (PMC11111980; doi:10.3389/frhs.2024.1304694)
Supplement: Supplementary file 1 [file Table1.docx]

**Search Strategy for Implementation Facilitation Literature Collection**

Citation databases:

PubMed, CINAHL, Thompson Scientific Web of Science (contains Science Citation Index, the Social Sciences Citation Index and the Arts and Humanities Index)

Inclusion criteria:

English language

Peer-review journals

| Facilitation | mp = keyword  MeSH = mapped subject heading  * = explosion of subject heading |
| --- | --- |
| AND |  |
| behavior change (mp)  best practice (mp)  change agents (mp)  coaching (mp)  “Diffusion of Innovation (MeSH)*  dissemination (mp)  evidence-based practice (mp)  evidence-based quality improvement (mp)  evidence uptake (mp)  external facilitation (mp)  facilitation role (mp)  facilitation skills (mp)  facilitation studies (mp)  fidelity (mp)  guideline implementation (mp)  implementation (mp)  implementation research (mp)  implementation science (mp)  implementation strategy (mp)  knowledge transfer (mp)  knowledge exchange (mp)  knowledge translation (mp)  Nursing Practice, Research-Based (MeSH)*  organizational change (mp)  practice coaching (mp)  practice facilitation (mp)  Practice Guidelines (MeSH)*  quality improvement (mp)  quality improvement initiatives (mp)  research implementation (mp)  research use (mp)  research utilization (mp)  system redesign (mp)  technical assistance (mp) | OR |
